# Guillain–Barre syndrome following dengue fever and literature review

**DOI:** 10.1186/s13104-015-1672-0

**Published:** 2015-11-27

**Authors:** Dissanayake Mudiyanselage Priyantha Udaya Kumara Ralapanawa, Senanayake Abeysinghe Mudiyanselage Kularatne, Widana Arachilage Thilak Ananda Jayalath

**Affiliations:** Department of Medicine, University of Peradeniya, Kandy, Sri Lanka

**Keywords:** Dengue fever, Guillain–Barre syndrome, Facial nerve palsy, Sri Lanka

## Abstract

**Background:**

Dengue is an arboviral infection that classically presents with fever, joint pain, headaches, skin flush and morbilliform rashes. The incidence of neurological symptoms and complications in dengue varies from 1 to 25 % that include encephalopathy, Guillain–Barre syndrome (GBS), acute motor weakness, seizures, neuritis, hypokalaemic paralysis, pyramidal tract signs, and a few more. Dengue fever as an antecedent infection in GBS is uncommon.

**Case presentation:**

A 34-years-old Sri Lankan Sinhalese male presented with fever, headache and myalgia of 3 days and developed leucopenia and thrombocytopenia without evidence of haemoconcentration. The diagnosis of dengue fever was confirmed as he had positive dengue NS1 antigen test on the third day of fever. He made full recovery and was discharged after 4 days of hospital stay. Six days later, he presented with history of acute flaccid weakness of both lower limbs and upper limbs which was of progressive ascending nature. The electromyography had evidence of demyelinating neuropathy and cerebrospinal fluid showed albuminocytological dissociation. Subsequently, IgM for dengue virus was positive.

**Conclusion:**

Dengue is endemic in Sri Lanka. Post dengue Guillain–Barre syndrome is a potential neurological complications of this infection.

## Background

Dengue is a common human arbovirus infection globally [1, 2]. The World Health Organization (WHO) estimates an annual incidence of approximately 100 million infections, with approximately 500,000 people with dengue haemorrhagic fever (DHF) requiring hospitalization, and a large proportion of them being children [2].

As the incidence of dengue and DHF is increasing, unusual manifestations are on the rise, although they are being under reported due to the lack of awareness [3]. Neurological manifestations so far reported include depression, convulsions, encephalopathy, encephalitis, aseptic meningitis, intracranial haemorrhage, intracranial thrombosis, myelitis, mononeuropathies, polyneuropathies, hemifacial spasm, peripheral facial paralysis and Guillain–Barre syndrome (GBS) [3–5]. GBS or acute inflammatory demyelinating polyradiculopathy (AIDP) is a post infectious ascending, usually demyelinating, polyradiculoneuropathy accompanied by areflexia, motor paralysis, and elevated cerebro spinal fluid (CSF) total protein without pleocytosis [5–9].

Well established associations with GBS are recent infections with *Campylobacter jejuni*, Cytomegalo virus, Epstein–Barr virus, *Mycoplasma pneumonia*, HIV. But dengue fever (DF) as an antecedent infection in GBS is uncommon [5, 10, 11]. Several previous reports have described GBS in patients with dengue [3, 5, 12]. Most of these cases were children and a few cases of post-dengue GBS in adults [5]. Herewith we present an adult case of possible post-dengue GBS in Sri Lanka and this case calls for special attention because the dengue infection remains a serious public health problem in many countries and the actual incidence of neurological complications is not well reported.

## Case presentation

A 34-years-old Sri Lankan Sinhalese man was admitted in August 2014, with pain and numbness of both lower limbs for 3 days, followed by weakness of both lower limbs with numbness and pain of upper limbs for 1 day. On admission he was not able to walk as usual and had no difficulty in breathing or coughing. He could pass urine without any difficulty and did not have constipation. Ten days ago, he had been admitted to the same hospital with high grade continued fever of 3 days duration with generalized body ache, headache, nausea and retro-orbital pain. Then he had leucopenia, thrombocytopenia and positive dengue NS1 antigen in the blood. He had uneventful dengue fever without haemoconcentration and was discharged after 3 days in the hospital.

On examination, he was conscious, well oriented and had normal vital parameters. Cardiovascular, respiratory and abdominal system examinations were normal. The limb examination revealed hypotonia with reduced power in all four limbs, the lower limbs being most affected. The tendon reflexes were absent even with reinforcement. Sensory modalities were intact. His neck muscle power was normal and cranial nerves were normal. Provisionally, the diagnosis of ascending progressive polyneuropathy suggestive of GBS was made.

He had normal full blood count, erythrocyte sedimentation rate, C-reactive protein, serum electrolytes, renal function tests, liver function tests and chest X-ray. Serology for Hepatitis B, HIV were negative. Dengue IgM was positive. However an electrophysiological study revealed severe demyelinating polyneuropathy in the lower and upper limbs. Cerebro spinal fluid study showed albuminocytological dissociation [CSF appearance was clear, red blood cell: nil, 2 lymphocytes, protein 235 mg/dl, glucose 4.3 mmol/l (random blood sugar was 6.4 mmol/l).]. Gram stain CSF showed no bacteria. Finally a diagnosis of GBS associated with dengue fever was confirmed.

**Fig. 1:**
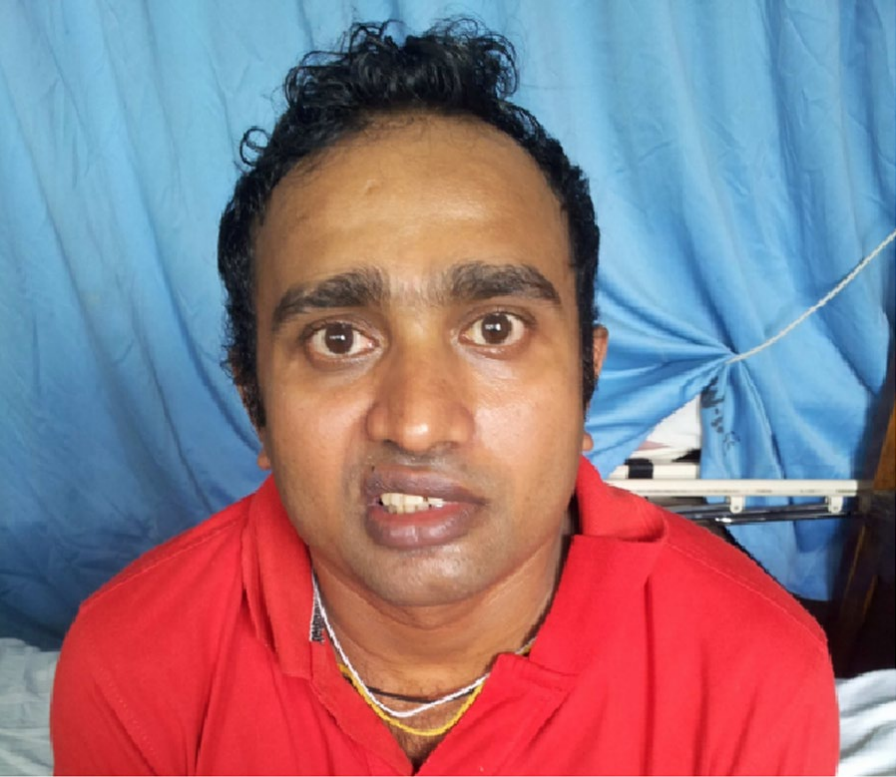
Second day of illness—left lower motor facial nerve palsy showing deviation of mouth to right

**Fig. 2:**
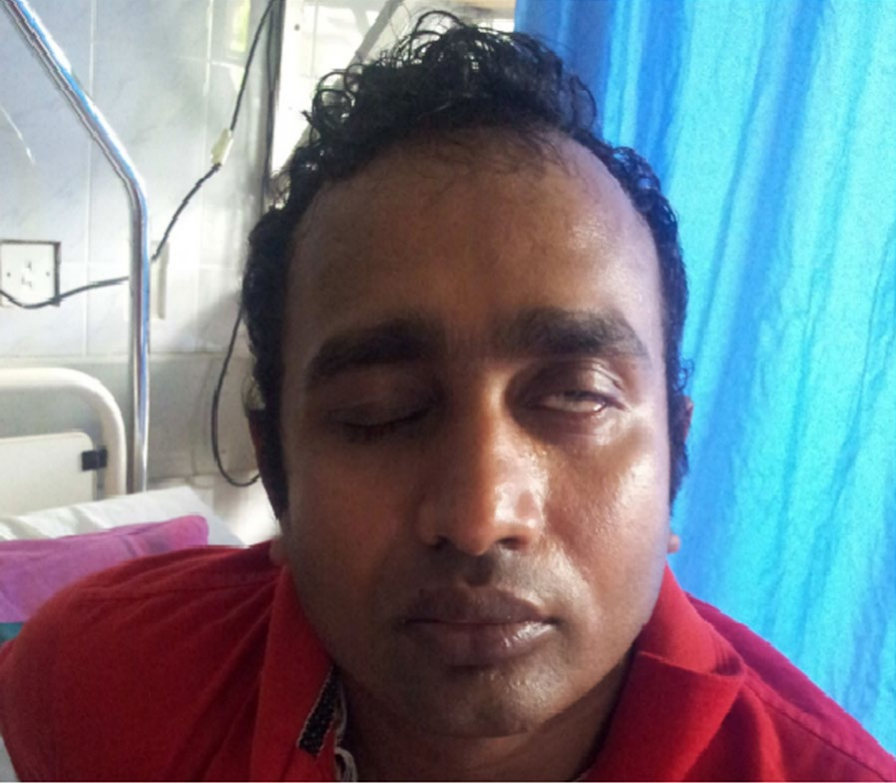
Second day of illness—partial closure of left eye with positive Bell’s sign and complete closure of right eye

**Fig. 3:**
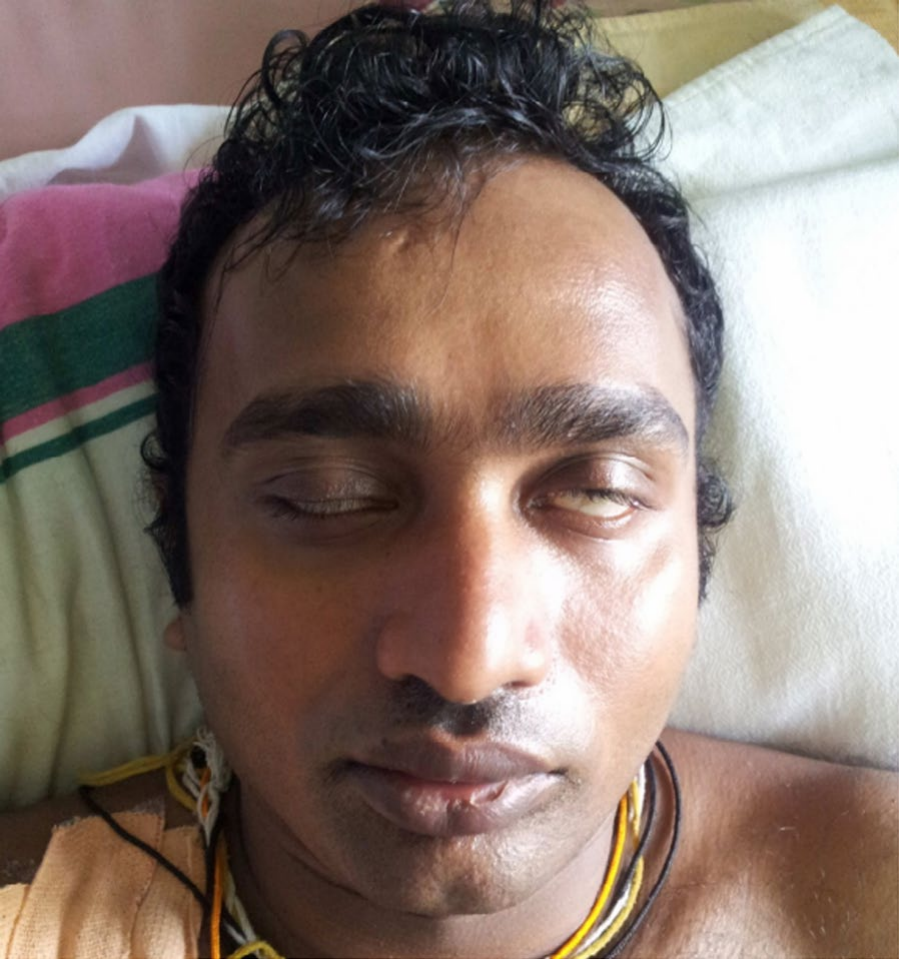
Fourth day of illness—showing bilateral lower motor facial nerve palsy with partial closure of both eyes and positive Bell’s sign

**Fig. 4:**
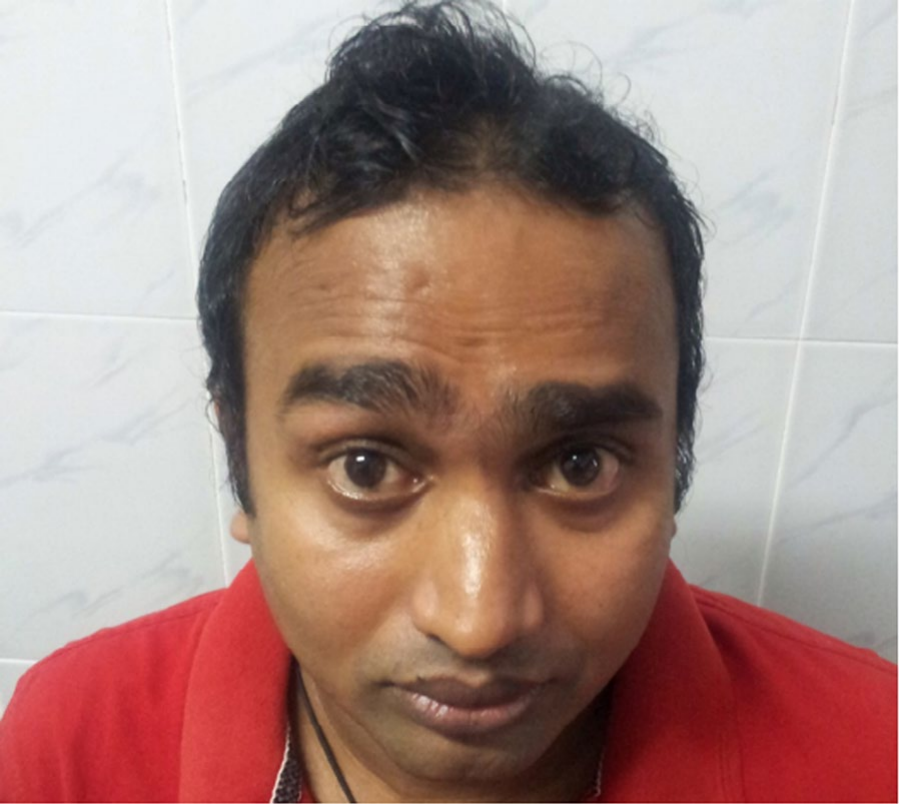
One month later—patient is attempting to raise his eyebrows showing improvement; right > left

**Fig. 5:**
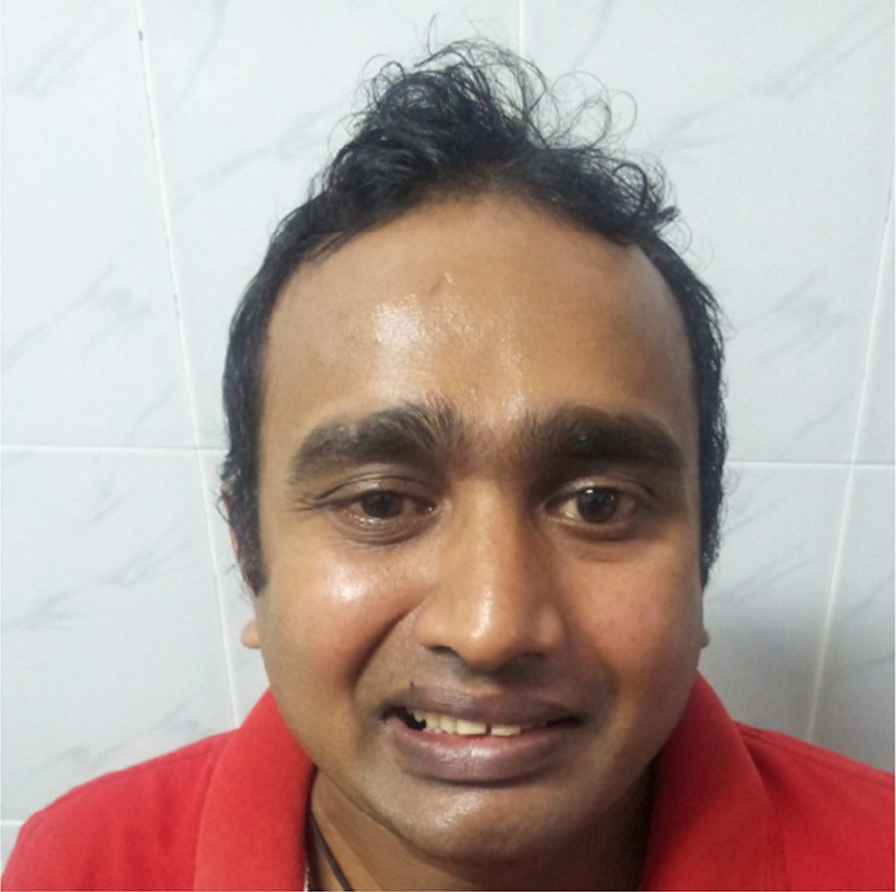
One month later—patient is attempting to show his teeth showing improvement; right > left

Therapeutically, plasmapheresis was carried out according to the protocol of the North American trial in which a total of 200–250 ml/kg is exchanged over 7–10 days. Meanwhile, on the second day, the limbs weakness increased and he developed left side lower motor type seventh cranial nerve palsy with positive Bell’s sign (Figs. 1, 2). But other cranial nerves, neck muscle power and respiration were normal. After two cycles of plasmapheresis on fourth day of hospital stay, he developed right side lower motor seventh cranial palsy with positive Bell’s sign (Fig. 3). After four cycles of plasmapheresis, there was little improvement of upper limbs weakness. After fifth cycle of plasmapheresis, his upper limbs improved further and right sided facial nerve weakness showed some improvement. After seventh cycle of plasmapheresis, his right facial nerve palsy completely improved and upper limbs and lower limbs showed significant improvement. Left side seventh nerve palsy showed significant improvement and other cranial nerves were normal. He developed neither urinary nor bowel incontinence. He did not develop autonomic symptoms such as resting tachycardia, gustatory sweating. With continued supportive measures, the patient made a significant recovery after 20 days of hospital stay and was referred to a rehabilitation hospital for further care. One month later we reviewed patient and by then he could walk alone. His facial nerves weakness also showed significant improvement (Figs. 4, 5).

## Discussion

Dengue, an arboviral infection that classically presents with fever, joint pains, headaches, skin flush and morbilliform rashes. Dengue viruses (DENV) belong to the family Flaviviridae and there are four serotypes of the virus referred as DENV-1, DENV-2, DENV-3 and DENV-4 [2]. All four serotypes can cause the full spectrum of disease including subclinical infection to a mild self limiting disease, dengue fever and severe disease that may be fatal including the dengue haemorrhagic fever/dengue shock syndrome (DHF/DSS).

Around 2.5 billion people around the world live in dengue endemic countries and are at risk of developing DF/DHF. Dengue is endemic in most countries of the South East Asia region where the detection of all four serotypes has now rendered these countries hyperendemic [2, 13, 14]. Dengue viral infections have become endemic in Sri Lanka since the mid 1960s, and since then the infections have increased dramatically over the past three decades [4, 13, 15].

We described this case scenario of GBS that had been preceded by a proven episode of dengue fever. Both episodes were not severe in their natural history as the patient did not develop plasma leak during the dengue infection and respiratory failure during subsequent GBS. The patient made recovery which was probably due to early use of plasmapheresis. Neurological manifestation associated with dengue fever has been reported from 25 different countries from Asia–Pacific, Americas, Mediterranean and Africa [5]. The involved ages had ranged from 3 months to 60 years in both sexes, being more common in children [5]. The incidence of neurological symptoms and complications among dengue patients varied from 1 to 25 % of all dengue admissions [2, 5, 12]. These manifestation included encephalopathy, GBS, acute motor weakness, seizures, neuritis, hypokalaemic paralysis, pyramidal tract signs, etc. [2, 5, 16].

There are a few previous reports that described GBS in patients with dengue particularly in adults. Gupta P et al. [17] reported a case of post-dengue GBS in a 24-year old unmarried male presenting with acute flaccid weakness of the lower limbs following a febrile illness of 3 days. The PCR for dengue was positive and IgM and IgG antibodies for DENV were found positive. The patient had been treated with intravenous human immunoglobulins and had a quick and complete recovery [17]. Similarly, Nee Kong Chew et al. [18] reported two cases of post-dengue GBS. The first case was a 43-year old woman with GBS presenting with the weakness of all four limbs and respiratory distress. She required assisted ventilation and immunomodulation treatment. The second case was a 51-year old man with bilateral facial weakness and numbness of extremities without motor weakness and recovered [18]. In 2012, Qureshi NK et al. reported a case of GBS following dengue fever in an adult patient. The case was a 39-year old female presented with acute flaccid weakness of both upper and lower limbs which developed in ascending and progressive fashion following a febrile illness of 3 days. She had positive IgM for DENV and was treated with intravenous immunoglobulins. In all these case reports, the onset of GBS occurred after recovery from the initial dengue infection similar to the present case report. The mechanism for post-dengue GBS is not fully understood. There is evidence that this is an immune-mediated neurological disease [17, 18]. Pro-inflammatory substances that participate in immune response to DENV such as TNF, complement, interleukins may have important role in the pathogenesis of GBS [19]. Immune response evoked by dengue fever may in turn cross-react with peripheral nerve components because of sharing of cross-reactive epitopes. This immune response can be directed towards the myelin or axon of peripheral nerves [20].

Several randomized clinical trials indicate that plasma exchange is more effective than supportive treatments in reducing the median time taken for recovery in GBS [5, 18]. Intravenous immunoglobulin (IVIg) appears as effective as plasma exchange and IVIg may be preferred because of its low side effect profile and ease of administration [5, 18]. Corticosteroids alone do not alter the outcome of GBS, and there is insufficient evidence to support their use in combination with immunoglobulin [5, 18]. Other treatments such as CSF filtration remain experimental and unproven [5]. Our patient was treated with seven sessions of plasma exchange and showed gradual improvement.

In our patient, the presence of a positive dengue-specific IgM antibody test indicates acute dengue illness. Neurological manifestations, pattern of electrophysiological study and the typical CSF findings were consistent with the diagnosis of GBS. Development of GBS in about 10 days after the initial manifestation of dengue support the association of these two conditions. Verma et al. shows that the patient presents with neurological syndromes like myelitis, myositis, GBS etc., dengue infection should be kept in differential diagnosis and should be ruled out especially during dengue outbreaks [21].

## Conclusion

Dengue is an endemic infection in Sri Lanka causing great impact on the health of the island nation. Other than encephalopathy, its various neurological complications have rarely been addressed. Acute flaccid paralysis or GBS is an uncommon neurological sequel of dengue fever and has not been previously reported from Sri Lanka. Though there are well established preceding infective agents causing GBS, preceding dengue fever as aetiological factor for GBS is not well documented. Thus our case report, as some shown in previous reports, call attention to the possibility that GBS may occur in association with dengue.

## Consent

Written informed consent was obtained from the patient for publication of this case report and accompanying images.

## Abbreviations

DF: dengue fever
DHF: dengue haemorrhagic fever
DSS: dengue shock syndrome
DENV: dengue virus
GBS: Guillain–Barre syndrome
SEA: South east Asia
CSF: cerebro spinal fluid
ESR: erythrocyte sedimentation rate
CRP: C-reactive protein
RBC: red blood cells
